# There Is a Positive Dose-Dependent Association between Low-Dose Oral Minoxidil and Its Efficacy for Androgenetic Alopecia: Findings from a Systematic Review with Meta-Regression Analyses

**DOI:** 10.1159/000525137

**Published:** 2022-06-30

**Authors:** Aditya K. Gupta, Deanna C. Hall, Mesbah Talukder, Mary A. Bamimore

**Affiliations:** ^a^Mediprobe Research Inc., London, Ontario, Canada; ^b^Division of Dermatology, Department of Medicine, University of Toronto School of Medicine, Toronto, Ontario, Canada

**Keywords:** Alopecia, Minoxidil, Meta-regression, Dosage

## Abstract

**Background:**

Recently, low-dose oral minoxidil (LDOM) has entered the landscape of therapies for androgenetic alopecia (AGA). We determined whether using LDOM is associated with improving AGA in a dose-dependent manner; secondarily, we examined whether a dose-dependent association also exists for safety.

**Methods:**

Systematic searches were conducted in PubMed and Scopus to identify studies that would be eligible for our quantitative analyses; the logistics of our analyses was determined by the data we gathered.

**Results:**

Six studies were eligible for quantitative analyses; we conducted meta-regressions. We found that, for persons with AGA, increasing the dosage of LDOM by 1 mg/day was − after six months − significantly associated with an expected sex-adjusted increase in hair diameter (mean difference = 1.4 μm, *p* = 0.01), total hair density (mean difference = 47.1 hairs/cm<sup>2</sup>, *p* = 0.007), terminal hair density (mean difference = 9.1 hairs/cm<sup>2</sup>, *p* = 0.001), risk of hypertrichosis (mean difference = 17.9%, *p* = 0.006), and cardiovascular adverse events (mean difference = 4.8%, *p* = 0.004).

**Conclusions:**

Our study produced new evidence as our work is the first to show a positive dose-dependent association between the use of LDOM and change in hair diameter, hair density, risk of hypertrichosis, and cardiovascular adverse events for persons with AGA. Future randomized trials could produce causal evidence that would corroborate these dose-dependent associations.

## Introduction

Androgenetic alopecia (AGA) is the most common type of hair loss [1, 2], and having this condition is associated with the development of anxiety and depression [3]. Common therapeutic agents for AGA include topical minoxidil and the 5-alpha reductase inhibitors, namely dutasteride and finasteride [4, 5, 6]. Minoxidil − as Heymann [7] alluded to − has come a long way; this agent was first used orally as an antihypertensive before being used topically as a medicine for hair loss [7], and an untoward effect of the oral antihypertensive was hypertrichosis (i.e., excessive hair growth) [8]. With hypertrichosis, excessive growth can occur anywhere on the body − such as the eyebrows, face, forearms, and forehead [8, 9]. The observation of this side effect led to randomized trials that examined the efficacy of topical minoxidil solution for hair loss; the research endeavors culminated in 2% topical minoxidil solution being approved by the US Food and Drug Administration (FDA) to treat male and female AGA in 1988 and 1992, respectively [4].

Now, oral minoxidil has come back into the picture; recent research endeavors aim to determine whether alopecia can be treated with “low doses” of the oral agent. Conventionally, low-dose oral minoxidil (LDOM) refers to any dose below 5 mg [7]. We systematically reviewed the peer-reviewed literature and quantitatively synthesized the evidence base to determine whether the efficacy and safety of oral minoxidil are correlated with its dosage.

## Materials and Methods

On December 3, 2021, systematic searches were conducted in the PubMed and Scopus databases. Studies that were eligible for quantitative analyses were trials that were published in the English language and investigated the efficacy of monotherapy with oral minoxidil in persons diagnosed with AGA. The logistics of our quantitative analyses was determined by the data that were gathered after the systematic search; all analyses were done with RStudio [10], and alpha (i.e., cutoff for statistical significance) was set at 5%.

## Results

**Fig. 1:**
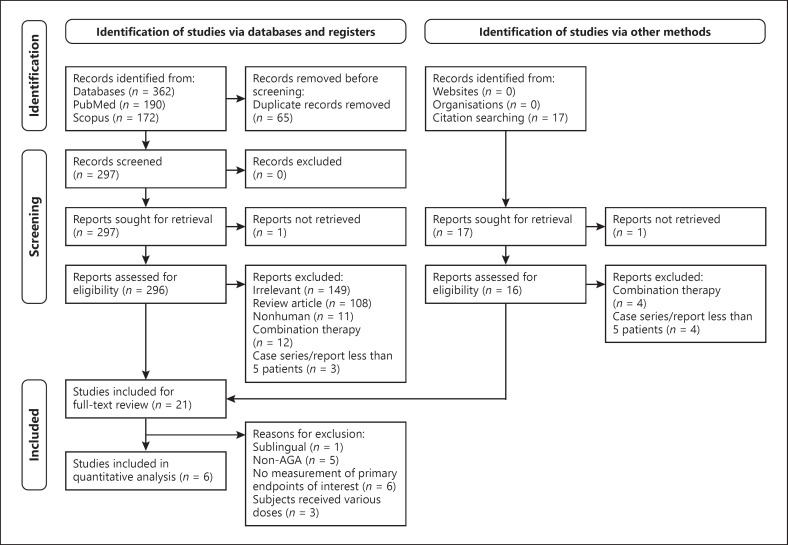
Identification of eligible studies for quantitative analyses. Flowchart depicting the exclusion processes for the identification of studies that will be included in our review and subsequent analyses.

Figure 1 delineates our search process; we identified six studies that were eligible for quantitative analyses [11, 12, 13, 14, 15, 16] (Fig. 1). For our four endpoints of interest, Table 1 indicates which of the six studies the respective endpoints used data from; across the eligible studies, the dosages ranged from 0.25 to 5 mg daily.

Our three primary endpoints were the 6-month change in hair density (total and terminal) and hair diameter. Our two secondary endpoints were safety-related ones, namely risk of hypertrichosis at 6 months and risk of cardiovascular adverse events within 6 months.

To determine whether increasing the dosage of oral minoxidil is significantly associated with an increase in hair diameter, hair density (total and terminal), risk of hypertrichosis, and risk of cardiovascular adverse events, we conducted an ecological regression for each endpoint (hence five regression analyses). Regressions produce regression coefficients (often denoted as “β”) − which are estimates of the expected change in outcome variable that results from a unit change in the explanatory variable. Our regressions were “ecological” because the unit of observation is from the group (i.e., aggregate) level − and not the individual (i.e., patient) level: each observation came from the oral minoxidil arm of the eligible study.

**Fig. 2:**
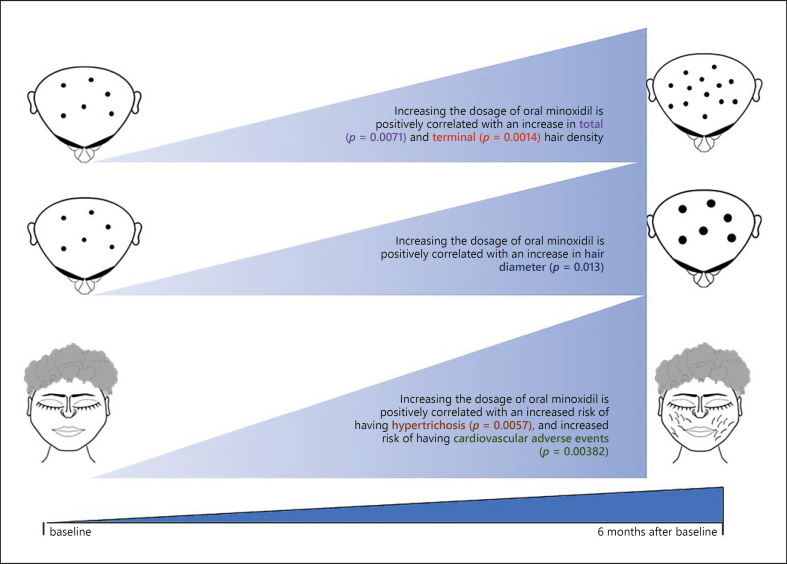
A depiction for the summary of our findings. There is a positive correlation between dosage of oral minoxidil and hair density (total and terminal), hair diameter, risk of hypertrichosis, and risk of cardiovascular adverse events.

Results from our robust sex-adjusted ecological regressions support that the efficacy and safety of oral minoxidil is positively correlated with dosage. Figure 2 summarizes the findings of the current study.

We found that, for persons with AGA, increasing the dosage of oral minoxidil by 1 mg daily is significantly associated with an expected increase of 47.1 and 9.1 hairs/cm^2^ in total (*p* = 0.0071) and terminal (*p* = 0.0014) hair density, respectively, after 6 months; the estimates are adjusted for sex differences (Table 2). Our results showed that, after 6 months, increasing the dosage of oral minoxidil by 1 mg daily is − on average − significantly associated with a sex-adjusted increase of 1.4 μm in hair diameter for persons with AGA (*p* = 0.013) (Table 2).

We found that, after adjusting for sex, increasing the dosage of oral minoxidil by 1 mg daily is, on average, significantly associated with an increased risk of having hypertrichosis and cardiovascular adverse events by 17.6% (*p* = 0.0057) and 4.8% (*p* = 0.00382), respectively, in persons with AGA after 6 months (Table 2). We deemed a cardiovascular adverse event to be an untoward outcome that is connected to abnormal cardiovascular physiology. Across the six studies that were eligible for our quantitative analyses, the cardiovascular adverse event we identified − as per our definition thereof − were hypotension, edema, increased hear rate, palpitation, and abnormal electrocardiogram.

## Discussion

We produced quantitative evidence for LDOM and its dose-dependent effect on hair diameter, hypertrichosis, cardiovascular adverse events, and hair density in persons with AGA. Minoxidil was inaugurally used as an oral antihypertensive agent [9, 17, 18, 19, 20]; it was used to treat severe high blood pressure. In early oral minoxidil trials for hypertension [18, 21], patients had their blood pressure significantly lowered and some coincidentally − or perhaps serendipitously − developed hypertrichosis [9, 17, 18]. In the clinical trial by Gottlieb et al. [18], five of the eight (i.e., 62.5%) patients in the oral minoxidil arm developed hypertrichosis [18]. Although subjects in the minoxidil arm were also on standard therapy with beta-adrenergic blockade, the hypertrichosis is attributable to minoxidil as the “untoward” effect was not reported for the hydralazine arm that also had patients on the standard therapy [18]. In 1975, Mehta and colleagues [21] reported the occurrence of hypertrichosis in 10 of the 17 (i.e., 58.8%) patients who participated in the single-arm trial on oral minoxidil. In the early minoxidil trials such as those by Mehta et al. [21] and Gottlieb et al. [18], the dose of minoxidil varied across subjects − albeit − the minimum dosage was not less than 10 mg daily. The hypotensive effect of oral minoxidil occurs at dosages above 10 mg daily, and this explains why the agent is used at dosages no greater than 5 mg daily for hair loss and hence the term “low-dose oral minoxidil” [7]. As mentioned earlier, the dosages in our quantitative analyses ranged from 0.25 mg daily to 5 mg daily.

Shortly after the reports on hypertrichosis in the 1970s, it became − as Bryan [22] put it − an “open secret” that oral minoxidil, an antihypertensive, promoted the growth of terminal hair. The commencement of trials that examined the therapeutic impact of topical minoxidil solution was buttressed by “promising” observations physicians encountered in clinical practice [22]: dermatologists observed that 40% of male patients on minoxidil experienced growth of terminal hair, while another 40% reported halting in hair loss; the remaining 20% of patients reported no impact on hair growth. Clinical trials that examined the efficacy of topical minoxidil solution on alopecia began around 1978 [22], and like, Heymann [7], many dermatologists were excited when the US FDA approved the treatment of AGA with 2% minoxidil solution for men (in 1988) and women (in 1992) [7].

After the 1990s, both the foam and solution forms of 5% topical minoxidil were approved for this condition [4, 19]. Itching and scaling were common untoward consequences of using topical minoxidil solution [19, 23]; hence, the foam was formulated to address this drawback of the solution as it is free of propylene glycol (i.e., an irritant that is a constituent of topical minoxidil solution) [23]. However, itching and scaling have also been reported with use of topical minoxidil foam [19, 23].

So, minoxidil − as Heymann [7] stated − has almost come “full circle” as trials examining the efficacy of LDOM for nonscarring alopecia have been ongoing recently such an agent would overcome the issue of allergic contact dermatitis (which the topical forms cause). Given that doses above 10 mg lower blood pressure [21], doses below 5 mg would − predictably − do not reduce blood pressure in normotensive individuals with hair loss.

Results from our meta-regression showed that increasing the dosage of LDOM was significantly associated with an increase in both total and terminal density; similarly, we found a positive dose-dependent association for hair diameter. These positive correlations parallel with the dose-dependent effect that has been established for topical minoxidil solution [24]. For instance, the randomized controlled trial by Olsen et al. [25] showed that the 48-week change in terminal hair density was significantly greater with 5% topical minoxidil solution compared to its 2% counterpart in men with AGA (mean difference = 5.90 hairs/cm^2^, *p* = 0.025); similarly, the randomized controlled trial by Lucky et al. [26] showed that for women with AGA, the 5% solution is more efficacious than the 2% solution (mean difference = 5.30 hairs/cm^2^, *p* = 0.031). Furthermore, our recent network meta-analyses for male AGA [27] corroborated the superiority of 5% topical minoxidil solution over the 2% concentration for the 48-week change in terminal hair density (mean difference = 5.89 hairs/cm^2^, *p* < 0.05) [5, 27]. Furthermore, results of our current work are congruent with those of our recent network meta-analyses [27]; our previous work showed that the 6-month change in terminal hair density, for male AGA, was − on average − greater with 5 mg daily oral minoxidil than with the 0.25 mg daily dosage (mean difference = 39.33 hairs/cm^2^, *p* < 0.05) [27].

Our meta-regression supports a dose-dependent relationship for untoward effects. We found a positive dose-dependent correlation between the LDOM and the risk of cardiovascular adverse events − a finding which corroborates the report by Beach et al. [28]; therein, the authors explained how minoxidil, a vasodilator, can provoke unfavorable cardiac outcomes. We also found that an increase in oral minoxidil by 1 mg daily is significantly associated with an increased likelihood of having hypertrichosis. This finding supports those of Jimenez-Cauhe et al. [29]. The authors' meta-analyses showed that the rate of hypertrichosis − in patients with various forms of alopecia − was positively correlated with dosage; 0.25 mg daily was associated with 6.7% of patients receiving hypertrichosis, while 5 mg daily was associated with a 56.1% risk (*p* < 0.001) [29]. Furthermore, results from the authors' binary logistic regression reported higher odds ratios with increasing dose: with a reference dosage of 0.25 mg daily, the odds ratio − for frequency of hypertrichosis − were 5.20, 12.82, and 17.72 with dosages of 1 mg, 2.5 mg, and 5 mg daily, respectively; the three odds ratios were statistically significant (*p* < 0.001) [29].

Our findings contribute to the dermatologic literature by corroborating previous works [5, 27, 29] and providing new evidence, our work is the first to show that, for individuals with AGA, the use of LDOM is associated with an increase in hair diameter, hair density, risk of hypertrichosis, and risk of cardiovascular adverse events in a dose-dependent manner. While LDOM may be a promising option for the condition, its safety profile for AGA − as Huang and Senna [5] stated − needs to be further elucidated with randomized evidence. Nevertheless, our findings support the conduct of large head-to-head trials which − as Villani et al. [17] put it − aimed to search for the perfect dose of oral minoxidil to treat AGA, and “coming full circle” could effect a “paradigm shift.”

## Statement of Ethics

This research did not directly involve human subjects; hence, approval from a Research Ethics Board was not required.

## Conflict of Interest Statement

The authors have no conflict of interest to declare.

## Funding Sources

Our work did not receive any funding.

## Author Contributions

Aditya K. Gupta was mainly responsible for conceptualizing this work, wrote the 1st draft of this manuscript, and provided critical revisions. Deanna C. Hall and Mesbah Talukder collected and extracted data. Mary A. Bamimore conducted all statistical analyses, wrote the 1st draft of the manuscript, and provided critical revisions.

## Data Availability Statement

To access data analyzed during this study, further inquiries can be directed to the corresponding author.

## Figures and Tables

**Table 1:** Trials − on AGA − used for data analyses of the four endpoints, namely 6-month change in hair diameter, risk of hypertrichosis, and hair density (terminal and total)

Studies used for quantitative analyses of the respective outcomes	Outcome (measured at baseline and after 6 months)			
	hair diameter	total hair density	terminal hair density	occurrence of hypertrichosis
Jha et al. [15]				✓
Panchaprateep and Lueangarun [11]	✓	✓	✓	✓
Pirmez and Salas-Callo [12]		✓	✓	✓
Ramos et al. [14]		✓	✓	✓
Vahabi-Amlashi et al. [13]	✓	✓		
Vastarella et al. [16]	✓	✓	✓	✓

**Table 2:** Positive correlation between the dosage of oral minoxidil and the 6-month change in hair diameter, risk of hypertrichosis, and hair density (total and terminal) − for persons with AGA

Outcome	Point estimate (β)	*p* value
Change in diameter after 6 months, µm	1.39	0.0130
Change in total hair density after 6 months, hairs/cm^2^	47.10	0.0071
Change in terminal hair density after 6 months, hairs/cm^2^	9.14	0.0014
Change in 6-month risk of cardiovascular adverse events,^†^ %	4.76	0.00382
Change in 6-month risk of hypertrichosis, %	17.85	0.0057

The point estimate (i.e., (3) corresponds to an estimate of the change in the outcome that results from a unit change in oral minoxidil dosage across persons with AGA; estimation accounts for sex difference. For example, the “β = 47.10” means the following: for persons with AGA, increasing the dose of oral minoxidil by 1 mg is − on average − associated with total hair density increasing by 47.10 hairs per cm^2^ after 6 months − after accounting for sex differences. EKG, electrocardiogram.

† Hypotension, edema, increased heart rate, palpitation, and abnormal EKG were identified as adverse events; and we counted these five outcomes as an occurrence of a cardiovascular adverse event.
